# Epidermal Growth Factor Protects Squamous Cell Carcinoma against Cisplatin-Induced Cytotoxicity through Increased Interleukin-1β Expression

**DOI:** 10.1371/journal.pone.0055795

**Published:** 2013-02-01

**Authors:** Shian-Chin Ko, Chi-Ruei Huang, Jiunn-Min Shieh, Jhen-Hong Yang, Wen-Chang Chang, Ben-Kuen Chen

**Affiliations:** 1 Department of Chest Medicine, Chi Mei Medical Center, Tainan, Taiwan, Republic of China; 2 Institute of Bioinformatics and Biosignal Transduction, College of Bioscience and Biotechnology, National Cheng Kung University, Tainan, Taiwan, Republic of China; 3 Department of Pharmacology, College of Medicine, National Cheng Kung University, Tainan, Taiwan, Republic of China; 4 Graduate Institute of Medical Sciences, College of Medicine, Taipei Medical University, Taipei, Taiwan, Republic of China; University of Manitoba, Canada

## Abstract

The expression of cytokines, such as IL-1β, and the activation of the epidermal growth factor receptor (EGFR) are crucial regulators in the process of carcinogenesis. The correlation between growth factor and activated cytokine signals in the control of tumor development is a critical issue to be clarified. In our study, we found that the IL-1β gene and protein expression were induced by EGF in squamous cell carcinoma. To clarify the mechanism involved in EGF-regulated IL-1β expression, we examined the transcriptional activity and mRNA stability of IL-1β in EGF-treated cells. We found that EGF induced the expression of IL-1β and was mediated through transcriptional activation, but not through mRNA stability. The involvement of Akt and NF-κB signaling pathways in the EGF-induced IL-1β gene expression was confirmed by knockdown of RelA and Akt in cells or treating cells with Akt and NF-κB inhibitors, LY294002 and parthenolide, respectively. The expression of dominant negative IκB also repressed the activation of NF-κB and inhibited EGF-induced IL-1β expression. Using immunofluorescence staining assay, the EGF-stimulated nuclear translocation of NF-κB (p65) was inhibited by pre-treating cells with LY294002 and parthenolide. Furthermore, EGF increased the binding of NF-κB to the NF-κB binding site of the IL-1β promoter through the activation of the Akt/NF-κB pathway, which resulted in activating IL-1β promoter activity. The expression and secretion of IL-1β induced by EGF considerably reduced chemotherapeutic drug cisplatin-induced cell death. These results showed that EGF enhanced the expression of IL-1β, which was mediated by the Akt/NF-κB pathway. The activation of EGF signaling and increase of IL-1β contributed to chemotherapeutic resistance of cancer cells, suggesting that the expression of IL-1β may be used as a biomarker to evaluate successful cancer treatment.

## Introduction

Chronic inflammation promotes the progression of normal cells to malignancy and supports the survival of various malignancies through the production of proinflammatory cytokines. Proinflammatory molecules, such as interleukin-1 (IL-1) and interferon-γ, can activate and recruit myeloid-derived suppressor cells (MDSC) to the tumor sites, resulting in strong suppression of various T-cell functions [1]–[3]. The IL-1 family consists of 2 proinflammatory cytokines (IL-1α and IL-1β, IL-1 receptor antagonist (IL-1Ra), and 2 receptors (the biologically active IL-1 receptor type I (IL-RI) and the inert IL-1RII) [4]. IL-1α and IL-1β are both pro-inflammatory cytokines that are synthesized as precursor molecules (pro-IL-1α and pro- IL-1β) by several cell types. Pro- IL-1α is biologically active and must be cleaved by calpain to generate smaller mature protein. By contrast, pro- IL-1β is biologically inactive and requires enzymatic cleavage by IL-1β-converting enzyme (ICE) or caspase-1 to become active. IL-1α is bound primarily to the membrane, whereas IL-1β is secreted and represents the predominant extracellular form of IL-1 [5]. A recent study showed that, in the absence of exogenous stimuli, a number of human cancer cells spontaneously produce functional IL-1β, which leads to constitutive activation of the inflammasome [6]. Secretable IL-1β, derived from the microenvironment or the malignant cells, activates inflammation that promotes invasiveness and induces tumor-mediated suppression [7], [8]. In the regulation of IL-1β expression, transcriptional activation and posttranscriptional regulation can mediate its expression. For example, the regulation of IL-1β mRNA stability through AU-rich elements (ARE) has been reported [9]. Pro- IL-1β synthesis is induced by LPS through activation of the NF-κB and MAPK pathways [10], [11]. The expression of IL-1β stimulates angiogenesis and facilitates tumor growth and metastasis in human cancer cells [12].

The epidermal growth factor (EGF) receptor signaling pathway regulates fundamental functions in cells, including survival, proliferation, and metastasis [13]. Activation or overexpression of EGFR is a common feature in various human cancers [14]. Overexpression and EGFR phosphorylation are frequently detected in several cancers, such as head and neck squamous cell carcinoma (HNSCC), and lung, breast, prostate, ovary, and bladder cancers [15]–[18]. Increased expression of ErbB receptors or ligands, such as transforming growth factor-α (TGFα), amphiregulin (AREG), neuregulin-1 (NRG1), and cripto-1 (TDGF-1), are associated with mammary hyperplasia and adenocarcinoma development [19]. The activation of EGFR signaling regulates the expressions of several genes that contribute to tumor development. For example, the cyclooxygenase-2 gene induced by EGF plays a crucial role in regulating EGF-induced tumorigenesis [20]. In addition, EGF also stimulates the expression of cytokine secretion and expression, such as IL-6 and IL-8 [21], [22]. EGF induces the expression of granulocyte colony-stimulating factor (G-CSF) and IL-6 in multipotential stromal cells or mesenchymal stem cells (MSCs), suggesting that EGF may play a role in MSC-mediated support of hematopoiesis in bone marrow [23]. EGF also enhances cell migration of ovarian carcinoma through the induction of IL-6 [22]. Although these results indicate that EGF-regulated physiological functions may be partially affected by the induction of cytokines, e.g. IL-8 and IL-6, the molecular mechanisms involved in EGF-induced IL-1β expression and the contribution of EGF-induced IL-1β to cellular functions remains unknown.

To clarify the potential cross-talk and feed-back regulation between tumor cells and cytokines in surrounding microenvironments, we investigated the expression of cytokines induced by EGF in squamous cancer cells. This study clarified the mechanism involved in the regulation of EGF-induced cytokines, such as IL-1β and identified its function in mediating cisplatin-induced cytotoxicity. Our results suggest that IL-1β can be considered the target for combination treatment with chemotherapeutic drugs, such as cisplatin, to treat EGFR signaling-activated squamous cancer.

## Materials and Methods

### Cell culture

Cell lines of human squamous cell carcinoma (A431) and human oral squamous cell carcinoma (SCC4 and SCC25) were purchased from American Type Culture Collection. Human oral cancer cells (TU183) and oral squamous cell carcinoma (CA922) were kindly provided by Dr. Kwang-Yu Chang (National Health Research Institutes, Taiwan)[24]. In general, the cell lines A431, TU183 and CA922 were grown at 37°C under 5% CO_2_ in 10-cm plastic dishes containing 10 ml of Dulbecco's modified Eagle's medium supplemented with 10% fetal bovine serum, 100 µg/ml streptomycin, and 100 units/ml penicillin. The cell lines SCC4 and SCC25 were grown at 37°C under 5% CO_2_ in 10-cm plastic dishes containing 10 ml of Dulbecco's modified Eagle's medium: Nutrient Mixture F-12 (DMEM/F-12) supplemented with nonessential amino acids, 400 ng/ml hydrocortisone, 0.5 mM/ml sodium pyruvate, 10% fetal bovine serum, 100 µg/ml streptomycin and 100 units/ml penicillin. In this series of experiments, cells were treated with 50 ng/ml EGF (Pepro Technology, Rocky Hill, NJ) in culture medium supplemented with 10% fetal bovine serum, unless stated otherwise.

### Plasmid construction

The DNA fragment bearing the promoter region of IL-1β (987 bp) was generated by PCR and subcloned into pGL3 basic vector (Promega) at the KpnI and BglII sites. The forward and reverse primers were 5′-GACCTGTCAAAGAGGCAAAGGAGGG-3′ and 5′-TGTGCCTTGTGCCTCGAAGAGG-3′, respectively. The vector sequence was confirmed by DNA sequencing. Dominant negative IκB mutant was generated by N-terminal deletion of residues 1-45 using a standard PCR approach [25]. pTK minimal promoter with 5 repeated NF-κB biding sites was generated by PCR and subcloned into pGL3 basic vector (Promega).

### Immunofluorescence

Cells grown on chamber slides were treated with/without 50 ng/ml EGF for 1 h either alone or after 1 h pre-incubation with 20 µM LY294002 or 20 µM parthenolide. Cells were fixed with 4% paraformaldehyde (Sigma) in phosphate-buffered saline at 4°C for 10 min. The cells were then rinsed with phosphate-buffered saline three times and permeabilized with 1% Triton X-100 for 7 min. Next, the cells were pretreated with 1% bovine serum albumin in phosphate-buffered saline at 25°C for 60 min and incubated with indicated antibody at a dilution of 1∶100 for 1 h and treated with fluorescein isothiocyanate-conjugated donkey anti-rabbit IgG polyclonal antibodies (Jackson ImmunoResearch Laboratories, West Grove, PA) at a dilution of 1∶250 for 1 h. Finally, the cells were washed with phosphate-buffered saline, mounted in 90% glycerol containing 4,6-diamidino-2-phenylindole (Invitrogen), and examined by using a microscope (Olympus BX51).

### Transfection of cells with plasmids and luciferase assay

Transient transfection of cells with plasmids was performed with Lipofectamine 2000 (Invitrogen) according to the manufacturer's instructions but with slight modifications. A431 cells were replated 36 h before transfection at a density of 3 × 10^5^ cells in 2 ml of fresh culture medium in a 3.5-cm plastic dish. For use in transfection, 2 µl of Lipofectamine 2000 was incubated with 0.5 µg of pIL-1β/pGL3 luciferase plasmid in 1 ml of Opti-MEM medium for 30 min at room temperature. Cells were transfected by changing the medium with 1 ml of Opti-MEM medium containing the plasmids and Lipofectamine 2000 and then incubated at 37°C in a humidified atmosphere of 5% CO_2_ for 24 h. Following the change of Opti-MEM medium to 2 ml of fresh culture medium, cells were incubated further for an additional 24 h unless stated otherwise. The luciferase activity in cell lysate was determined as described previously [26].

### RNA interference

RNA interference vectors used in this study were obtained from the National RNAi Core Facility in the Institute of Molecular Biology, Academia Sinica (Taipei, Taiwan) as shown in follows: pLKO.1-shRNA-RelA- #TRCN0000014683 (target sequence, 5-GCCTTAATAGTAGGGTAAGTT-3); pLKO.1-shRNA-Akt1- #TRCN0000010162 (target sequence, 5-GGACAAGGACGGGCACATTAA-3); pLKO.1-shRNA- IL-1β- #TRCN0000058384 (target sequence, 5-GCGATTTGTCTTCAACAAGAT-3).

### Reverse transcription-PCR and quantitative real-time RT-PCR

Total RNA was isolated using the TRIzol RNA extraction kit (Invitrogen), and 1 µg of RNA was subjected to reverse transcription-PCR with ImProm-II™ (Promega). The IL-1β specific primers (sense, 5′-ATGGGATAACGAGGCTTATGTG-3′; antisense, 5′-CAAGGCCACAGGTATTTTGTC-3′), IL-8 specific primers (sense, 5′-GACAAGAGCCAGGAAGAAACC-3′; antisense, 5′-CTTTAGCACTCCTTGGCAAAAC-3′), IL-11 specific primers (sense, 5′-GCTGCACCTGACACTTGACT-3′; antisense, 5′-CTCACGGAAGGACTGTCTCTAA-3′), Akt1 specific primers (sense, 5′-GGAGGGCTCTGGACTCCCGTT -3′; antisense, 5′-CCACGTCGCTCATGGTGCCC-3′), RelA specific primers (sense, 5′-AGCAGCGTGGGGACTACGAC-3′; antisense, 5′-AGGCTGGGGTCTGCGTAGGG-3′), IκB specific primers (sense, 5′-ATGGTCAAGGAGCTGCAGGAGATC-3′; antisense, 5′-TCATAACGTCAGACGCTGGCCTC-3′) and glyceraldehyde-3-phosphate dehydrogenase primers (sense, 5′-CCATCACCATCTTCCAGGAG-3′; antisense, 5′-CCTGCTTCACCACCTTCTTG-3′) were used. The PCR products were separated by 2% agarose gel electrophoresis and visualized with ethidium bromide staining. For the quantitative real-time RT-PCR, cDNA synthesis was performed using the TITANIUM One-Step TR-PCR kit (Clontech) containing SYBR Green I. Real-time fluorescence monitoring and the melting curve analysis were performed with LightCycler according to the manufacturer`s recommendations. Negative controls containing no RNA template were included in each experiment. A melting curve was created at the end of the PCR cycle to confirm that a single product has been amplified. Data were analyzed by LightCycler software version 3.5 (Roch Molecular Diagnostics) to determine the threshold cycle (Ct) above the background for each reaction. The relative transcript amount of the target gene, calculated using standard curves of serial RNA dilutions, was normalized to that of glyceraldehydes-3-phosphate dehydrogenase (GAPDH) of the same RNA.

### Preparation of nuclear extracts

Cells from 10-cm plastic dishes were washed twice with PBS and scraped in 500 µl of PBS. Cells were collected by centrifuging at 7,500x*g* for 20–30 s, resuspended in 400 µl of buffer A (10 mM Hepes (pH 7.9), 1.5 mM MgCl_2_, and 10 mM KCl) and stand on ice for 10 min. Nuclei were pelleted by centrifugation at 7,500x*g* for 20–30 s. Pellets were resuspended in 100 µl of buffer C (20 mM Hepes (pH 7.9), 1.5 mM MgCl_2_, 0.2 mM EDTA, 420 mM NaCl, and 25% (v/v), glycerol) and stood on ice for 20 min. The suspension was centrifuged at 7,500x*g* for 2 min. The supernatants were collected and stored at −70°C until used. Buffer A and buffer C contained 0.5 mM dithiothreitol, 2 µg/ml of leupeptin, 1 mM orthovanadate, 2 µg/ml of pepstatin A, and 0.5 mM phenylmethylsulfonyl fluoride.

### Western blotting

The cytoplasmic fractions and nuclear extracts of cells were prepared for Western blot analysis according to the method described [27]. An analytical 10% SDS-PAGE was performed, and 30 µg of protein of each was analyzed, unless stated otherwise. For immunoblotting, proteins in the SDS gels were transferred to a polyvinylidene difluoride (PVDF) membrane by an electroblot apparatus. Antibodies against Akt, Phospho-Akt Ser473 (Cell Signaling techology®), NF-κB p65 C-20 (sc-372) and Ku70 (sc-12729) (Santa Cruz Biotechnology, Inc., Santa Cruz, CA), were used as the primary antibodies. Mouse or rabbit IgG antibodies coupled to horseradish peroxidase were used as secondary antibodies. An Immobilon™ Western Chemiluminescent HRP Substrate (Millipore) was used for detection.

### Enzyme-linked immunosorbent assay

Quantitation of the secretion of IL-1β to the culture medium was achieved by an Enzyme-linked immunosorbent assay (ELISA) according to the manufacturer's (R&D Systems). Briefly, after EGF treatment, 100 µl of culture medium was collected and incubated with pre-coated capture antibody at room temperature in a 96-well microplate. After washing step, 100 µl of detection antibody was added and incubated for another 2 h at room temperature. Add 100 µl of the working dilution of Streptavidin-HRP to each well and incubated for 20 min under protecting from light. After washing step, 100 µl of substrate solution was added and incubated for 20 min under protecting from light. After adding 50 µl of stop solution to each well, gently tapped the plate to ensure thorough mixing and used a microplate reader to quantify the optical density at 450 nm immediately.

### DNA affinity precipitation assay

Quantitation of the change of NF-κB binding to the IL-1β promoter element was achieved by a DNA affinity precipitation assay according to the method previously described [28]. In brief, 5′-biotinylated oligonucleotides and corresponding to the sense –315 to –286 bp and antisense strands of the IL-1β promoter element were annealed. The DNA affinity precipitation assay was performed by incubating 2 µg of biotinylated DNA probe with 200 µg of nuclear extract and 20 µl of streptavidin-agarose beads in phosphate-buffered saline at room temperature for 1 h with rotation. Beads were collected and washed three times with cold phosphate-buffered saline. The binding proteins were eluted by loading buffer and separated by SDS-PAGE, followed by Western blot analysis probed with specific antibodies.

### Cell counting Kit-8 (CCK-8) assay

Quantitation of the viability of cisplatin-treated cells was achieved by a Cell Counting Kit-8 assay (CCK-8) according to the manufacturer's (26992, Sigma-Aldrich). Briefly, 5000 cells were dispensed to each well of 96-well plate. After cells were treated with cisplatin for 24 h, 10 µl of CCK-8 solution was added into each well for additional 4 h. The absorbance at 450 nm was measured using a microplate reader.

### Apoptosis analysis

Quantification of apoptosis induced by cisplatin was performed with Annexin V and Propidium Iodide (PI) staining according to the manufacturer's (BioVision). Briefly, 1×105 cells were resuspend in Annexin V binding buffer and stained with Annexin V-PE and Propidium Iodide (1 µg/ml). After incubation at room temperature, the apoptotic cell was quantification by flow cytometry (BECKMAN COULTER Cell Lab Quanta™ SC).

## Results

### EGF regulates IL-1β expression in squamous cell carcinoma cell lines

**Figure 1:**
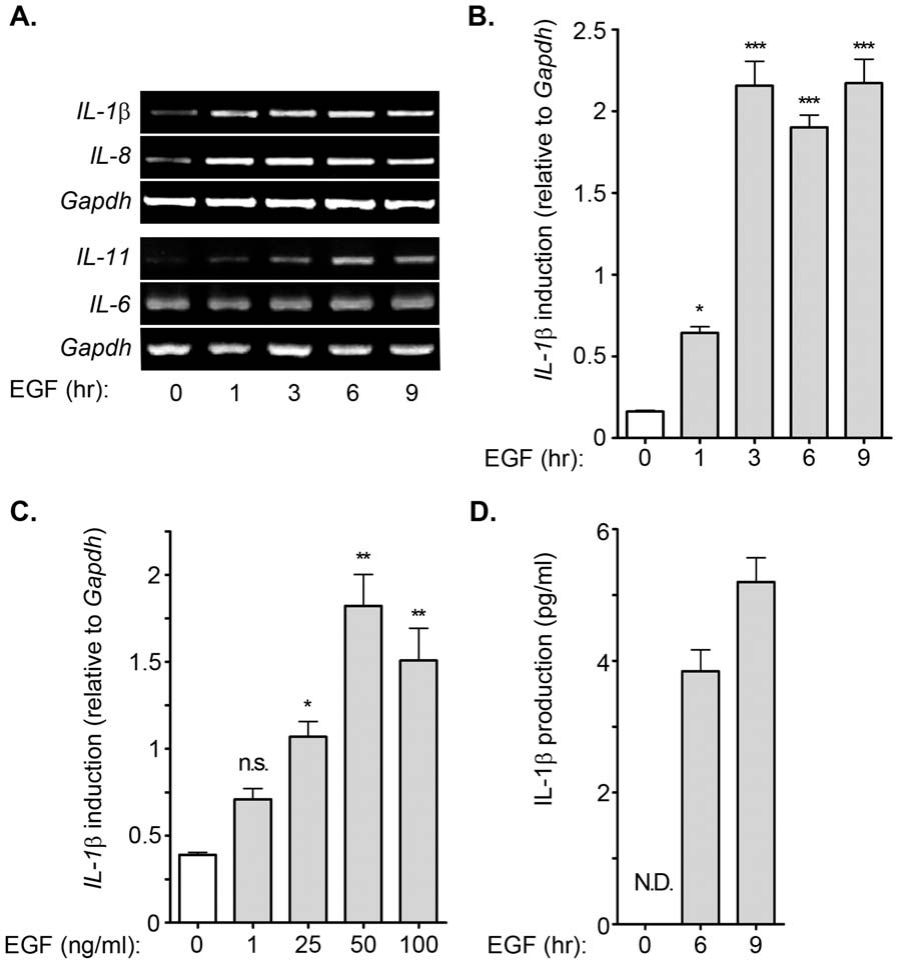
EGF induces the expression of IL-1β. (**A**) A431 cells were treated with 50 ng/ml EGF for a period of time (1∼9 h) before the extraction of RNA. The expression of IL-1β, IL-8, IL-11 and GAPDH mRNA was analyzed by RT-PCR and examined in 2% agarose gel. (**B and C**) A431 cells were treated with 50 ng/ml EGF for a period of time (1∼9 h) and various concentrations of EGF (1∼100 ng/ml). Expression of IL-1β was analyzed by Real-time PCR. The induction fold of EGF-induced IL-1β expression was normalized by GAPDH. (**D**) A431 cells were treated with 50 ng/ml EGF for 6 and 9 h, and then the condition media were collected for analyzing the IL-1β protein by ELISA. Values represent means ± S.E. of three independent experiments. ***: P<0.005; **: P< 0.01; *: P<0.05. N.D.: non-detected; n.s.: no significant difference.

**Figure 2:**
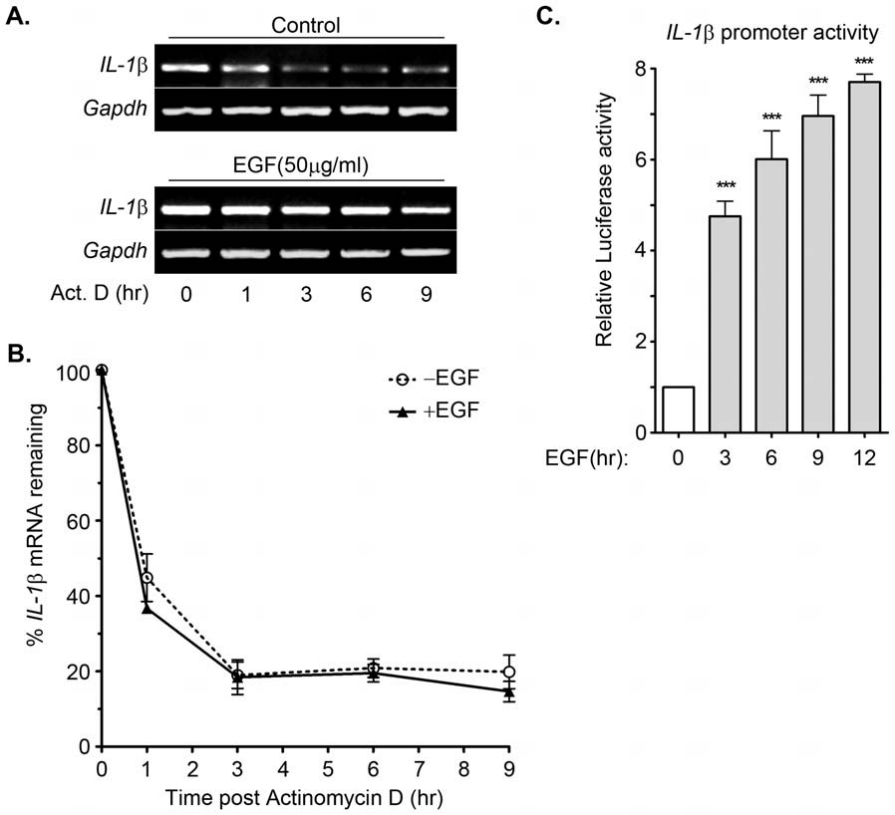
Effect of EGF on IL-1β mRNA stability and transcriptional activation. (**A and B**) A431 cells were treated with 50 ng/ml EGF for 3 h and then treated with 5 µg/ml actinomycin D for a period of time (1∼9 h). The expression of IL-1β and GAPDH mRNA was analyzed by RT-PCR and examined in 2% agarose gel. The expression of IL-1β mRNA was also analyzed by Real-time PCR. Relative levels of IL-1β were normalized by GADPH and the degradation rate was carried out by comparing to 0 h (as 100%). (**C**) A431 cells were transfected with 0.5 µg IL-1β promoter construct by lipofection and then treated with 50 ng/ml EGF for various time as indicated. The luciferase activities and protein concentrations were then determined and normalized. Values represent means ± S.E. of three determinations.

In our previous study, we identified that EGF induced the expression of inflammatory genes, such as COX-2 [20]. We studied the induction of inflammatory cytokines in EGF-treated malignant cells to determine whether EGF plays a role in the induction of cytokines to regulate carcinogenesis. To clarify whether the expression of pro-inflammatory cytokines was induced by EGF, cells were treated with EGF for 1 to 9 h. The results suggest that IL-1β, IL-8 and IL-11, but not IL-6 were up-regulated during EGF treatment in A431 cells (Figure 1A). The quantitative RT-PCR results showed that the IL-1β mRNA level was substantially elevated and reached the peak after 3 h of EGF treatment (Figure 1B). EGF also induced IL-1β gene expression in squamous carcinoma cells, such as SCC4, SCC25, TU183, and CA922 (Figure S1). These results indicate that IL-1β was a downstream target of EGF signaling. To further confirm that the specificity of EGF was involved in the regulation of IL-1β, we treated cells with various concentrations of EGF. The results showed that EGF induced IL-1β mRNA expression in a dose-dependent manner, however, the maximum effect of EGF on IL-1β mRNA induction was observed in 50 ng/ml EGF-treated cells (Figure 1C). In addition, EGF also increased IL-1β protein production and secretion in the cultured media (Figure 1D). To investigate whether the alteration of IL-1β mRNA stability was responsible for the EGF-induced IL-1β gene expression, a DNA-dependent RNA synthesis inhibitor, actinomycin D, was used to examine the mRNA stability in EGF-treated cells. Both parental and EGF-treated cells exhibited a similar mRNA degradation rate under the actinomycin D treatment condition (Figures 2A and 2B). We further studied the effect of EGF on IL-1β promoter activity using luciferase reporter assay. As shown in Figure 2C, EGF induced substantial IL-1β promoter activity in a time-dependent manner. These results showed that EGF stimulated the expression of IL-1β through transcriptional activation, but not the stabilization of mRNA in tumor cells.

### EGF induces IL-1β expression through PI3K/Akt and NF-κB dependent pathways

**Figure 3:**
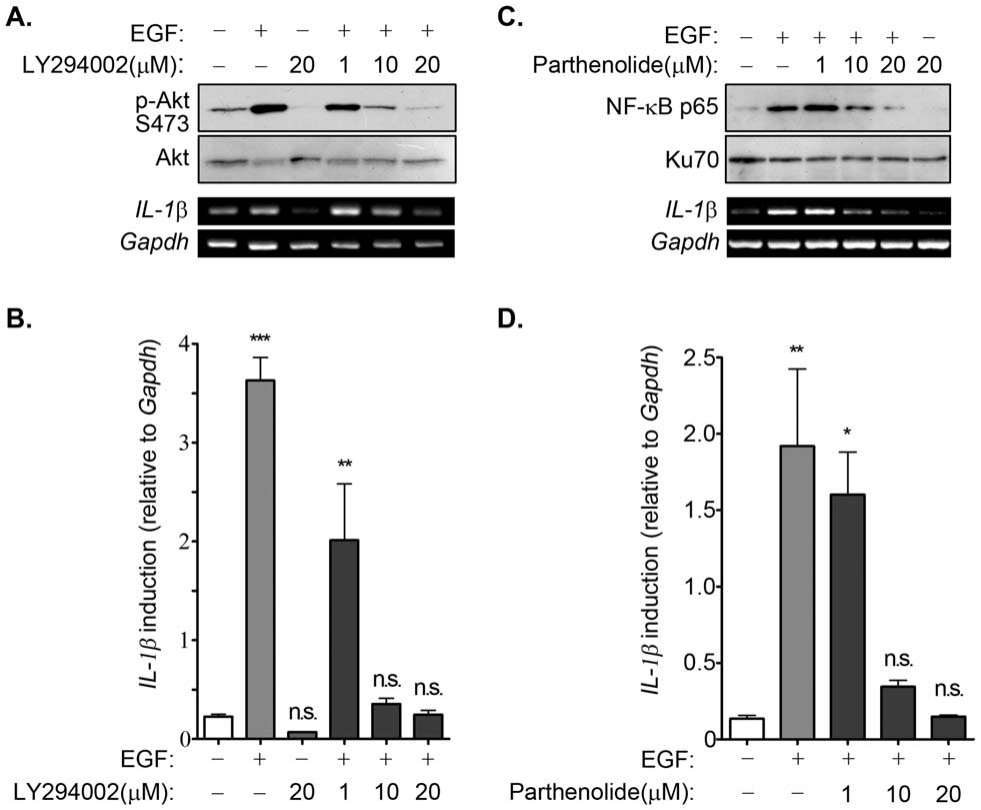
Activation of NF-κB is essential for EGF-induced IL-1β expression. (**A**) A431 cells were treated with various concentration of LY294002 as indicated for 1 h and followed by treated with 50 ng/ml EGF for 10 min (lysates) or 3 h (RNA). Lysates and RNA were prepared for examining the phosphorylation of Akt on serine 473 and IL-1βmRNA, respectively by using Western blot analysis and RT-PCR. (**B and D**) A431 cells were treated with various concentrations of LY294002 or parthenolide as indicated for 1 h, and followed by treated with 50 ng/ml EGF for 3 h. The expression of IL-1β was analyzed by Real-time PCR. The induction fold of EGF-induced IL-1β expression was normalized by GAPDH. (**C**) A431 cells were treated with various concentrations of parthenolide as indicated for 1 h and followed by treated with 50 ng/ml EGF for 1 h (nuclear extracts) or 3 h (RNA). Nuclear extracts and RNA were prepared for examining the nuclear translocation of NF-κB and IL-1βmRNA, respectively by using Western blot analysis and RT-PCR. Values represent means ± S.E. of three determinations. n.s.: no significant difference. ***: P<0.005; **: P< 0.01; *: P<0.05.

**Figure 4:**
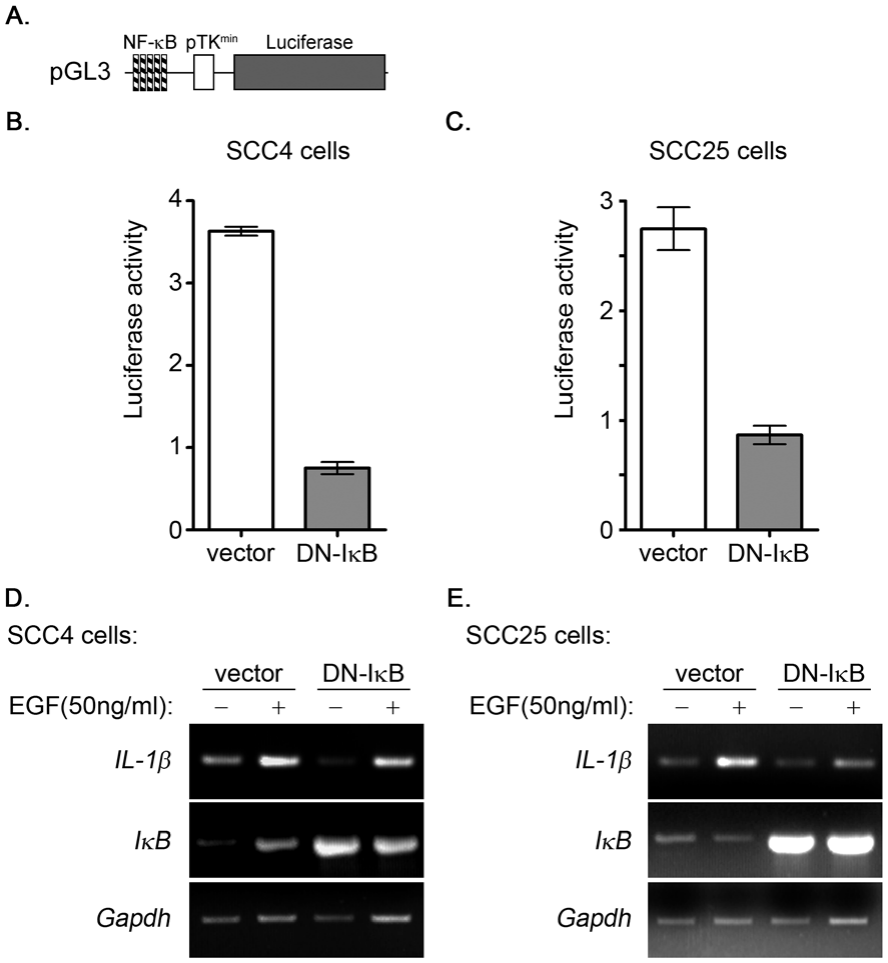
Dominant negative IκB inhibits the activation of NF-κB and reduces IL-1β expression in EGF-treated SCC4 and SCC25 cells. (**A**) The construct of pTK promoter with 5 repeated NF-κB biding sites bearing luciferase gene was presented. (**B and C**) SCC4 and SCC-25 cells were transfected with 0.5 µg pTK-NFκB promoter, 1 µg DN-IκB expression vector and 1 µg control vector by lipofection. The luciferase activities and protein concentrations were then determined and normalized. (**D and E**) SCC4 and SCC-25 cells were transfected with 1 µg dominant negative IκB (DN-IκB) expression vector or 1 µg control vector by lipofection and then treated with 50 ng/ml EGF for 6 h before the extraction of RNA. The expression of IL-1β, IκB and GAPDH mRNA was analyzed by RT-PCR and examined in 2% agarose gel.

Inhibitors that blocked EGF-activated JNK, ERK, PI3K/Akt, and NF-κB were used to clarify the signaling pathways involved in the regulation of EGF-induced IL-1β expression. As shown in Figure 3A, LY294002, which is an inhibitor of phosphoinositide 3-kinases (PI3Ks), inhibited EGF-induced Akt phosphorylation and IL-1β mRNA expression in a dose-dependent manner. We further confirmed these results using quantitative RT-PCR analysis, as shown in Figure 3B. These results indicated that EGF induces IL-1β mRNA expression through the PI3K/Akt pathway. Because PI3K played a role in the regulation of EGF-induced IL-1β gene expression, we investigated whether its downstream target, such as NF-κB which is a common mediator in cytokine signaling [29], also participated in the regulation of gene expression. Parthenolide, which is a sesquiterpene lactone that inhibits activation of the NF-κB pathway [30], inhibited EGF-induced activation of NF-κB and repressed EGF-induced IL-1β gene expression (Figure 3C). The quantitative RT-PCR results further confirmed the inhibitory effect of parthenolide on EGF-induced IL-1β induction (Figure 3D). To further confirm that PI3K/Akt and NF-κB pathways were involved in the regulation of EGF-induced IL-1β expression, stable cell lines with Akt1 or NF-κB knockdown via short hairpin (sh) RNA knockdown of Akt1 (shAkt1) and RelA (shRelA) were used. Indeed, EGF-induced the expression of IL-1β was inhibited in shAkt and shRelA cells as compared to parental cells (Figure S2). To study the effect of permanently prevent NF-κB activation on EGF-induced IL-1β expression, we utilized a dominant negative form of IκB (DN-IκB) that lacks all N-terminal phosphorylation sites, thus it is resistance to degradation but still with the ability of binding to NF-κB [25]. As shown in Figure 4, the overexpression of DN-IκB not only inhibited the activation of NF-κB (Figures 4B and 4C), but significantly reduced EGF-induced IL-1β expression (Figures 4D and 4E) in SCC4 and SCC25 cells. These results suggest that EGF-induced IL-1β expression occurred mainly through the PI3K/Akt and NF-κB pathways. We also studied the effects of JNK and ERK inhibitors, SP600125 and U0126, respectively on EGF-induced IL-1β gene expression. However, no significant effect of these inhibitors on IL-1β expression was observed (Figures S3A and S3B).

### EGF induces the nuclear translocation of NF-κB and its binding to NF-κB site on the IL-1β promoter

**Figure 5:**
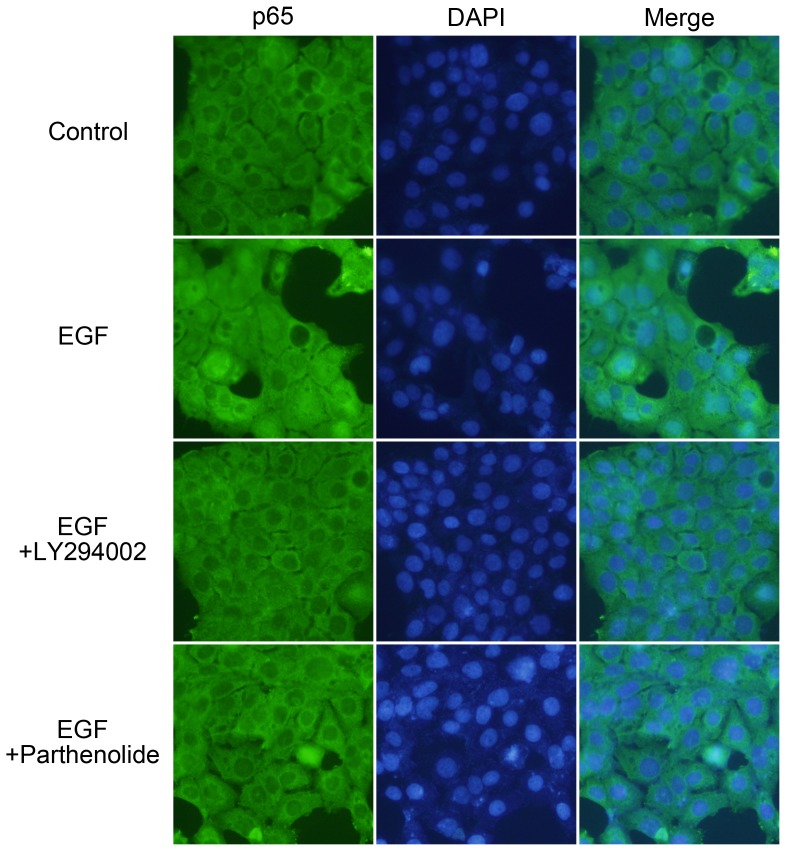
EGF enhances nuclear translocation of NF-κB. A431 cells were treated with 20 µM LY294002 and 20 µM parthenolide for 1 h and followed by treating with 50 ng/ml EGF for 1 h. Anti-p65 antibodies and DAPI were used for staining the nuclear translocation of NF-κB (p65) and DNA, respectively in immunofluorescence analysis.

**Figure 6:**
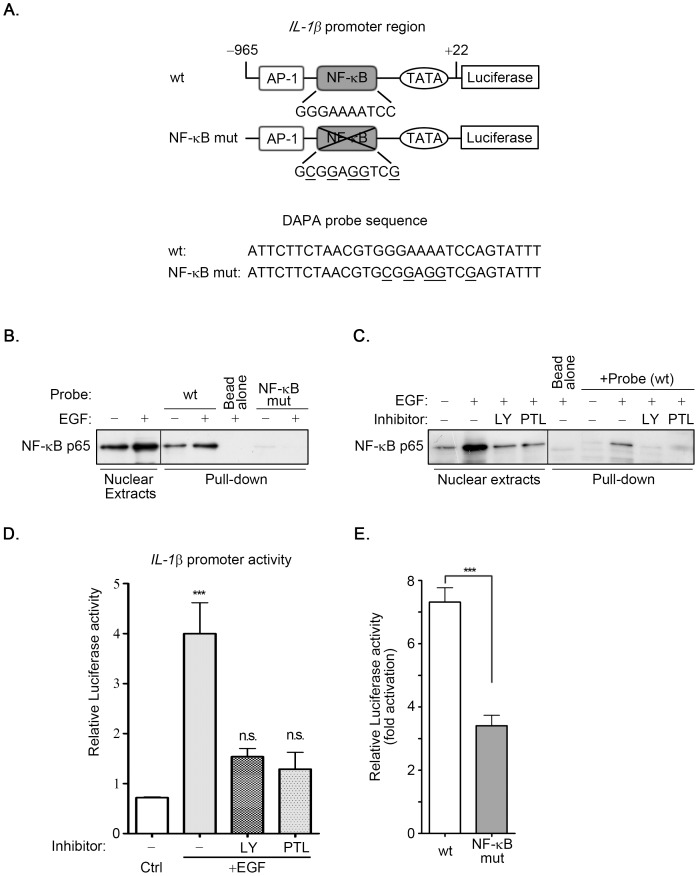
NF-κB binding site located on IL-1β promoter is essential for EGF-induced promoter activity. (**A**) Constructs of IL-1β promoter or mutation sequence of NF-κB binding site bearing luciferase gene were presented. (**B and C**) Nuclear extracts (NE) from A431 cells were treated with 20 µM LY294002 (LY) and 20 µM parthenolide (PTL) for 1 h, and followed by treating with 50 ng/ml EGF for 1 h. DNA affinity precipitation assay was performed as described under “Materials and Methods”. Binding of NF-κB p65 to wild-type probes, wt was analyzed by Western blot. The mutant sequence, NF-κB mut was used to serve as a negative control for wild-type sequence. (**D**) Cells were transfected with 0.5 µg IL-1β promoter construct by lipofection and then treated with 20 µM LY294002 (LY) and 20 µM parthenolide (PTL) for 1 h, and followed by treating with 50 ng/ml EGF for 6 h. The luciferase activities and protein concentrations were then determined and normalized. (**E**) Cells were transfected with 0.5 µg wild type (wt) or mutant (NF-κB mut) IL-1β promoter construct by lipofection and then treated with 50 ng/ml EGF for 6 h. The luciferase activities and protein concentrations were then determined and normalized. Values of luciferase activities are means ± S.E. of three determinations. ***: P<0.005; n.s.: no significant difference; Ctrl: control.

After responding to the survival signal in the cell, IκB was phosphorylated by IκB kinase (IKK), resulting in the decrease of IκB through the ubiquitin-proteasome-dependent degradation pathway. This event triggered the release of NF-κB from cytosol into the nucleus to elicit transcriptional activation through the binding of NF-κB to its response element [31]. Our results show that the PI3K/Akt and NF-κB pathways were involved in EGF-induced expression of IL-1β (Figures 3 and 4; Figure S2). Subsequently, we investigated whether the nuclear translocation of NF-κB was required for the induction of IL-1β expression in EGF-treated cells. As shown in Figure 5, EGF induced nuclear accumulation of NF-κB p65. Both LY294002 and parthenolide inhibited EGF-induced nuclear accumulation of NF-κB p65. To further verify that the binding of NF-κB to the promoter was essential for the regulation of IL-1β mRNA induction, the promoter region of IL-1β bearing the mutated NF-κB binding site (NF-κB mut) was sub-cloned to the luciferase-based reporter system. The mutated NF-κB binding was performed using the site-directed mutagenesis strategy (Figure 6A). In addition, DNA affinity precipitation assay was conducted to investigate whether the binding of NF-κB to the promoter region of the IL-1β gene was induced by EGF. As shown in Figure 6B, EGF enhanced nuclear accumulation and the binding of NF-κB p65 to the promoter. However, NF-κB lost the binding affinity with the NF-κB mutated probe (Figure 6B). These results indicated that the EGF-enhanced binding of NF-κB to the IL-1β promoter was achieved through the NF-κB binding site. We then studied the requirement of NF-κB in the regulation of EGF-induced IL-1β promoter activity. As shown in Figures 6C and 6D, both LY294002 and parthenolide inhibited the EGF-induced accumulation of NF-κB in the nucleus and the binding of NF-κB to the IL-1β promoter, and also inhibited EGF-induced IL-1β promoter activity. Furthermore, the EGF-induced promoter activity was substantially reduced in NF-κB mut (Figure 6E). These results showed that EGF stimulated the nuclear translocation of NF-κB to bind to the IL-1β promoter through the NF-κB binding site, resulting in transcriptional activation of gene expression.

### EGF-induced IL-1β reduces the cisplatin-induced cytotoxicity

**Figure 7:**
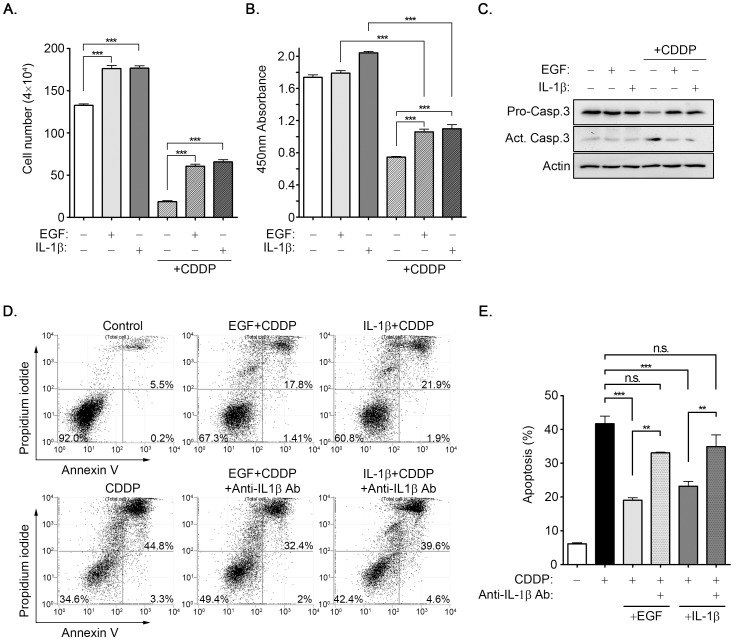
EGF-induced IL-1β reduces cisplatin-induced cytotoxicity. (**A-C**) A431 cells were treated with 50 ng/ml EGF and 5 ng/ml IL-1β for 6 h and followed by treating cells with 20 µM cisplatin (CDDP) for 24 h. Cell number and viability were analyzed by using cell counter (**A**) and the CCK-8 assay (**B**), respectively. Lysates were prepared and subjected to Western blot with antibodies against caspase-3 and actin (**C**). (**D and E**) Cells were treated with 50 ng/ml EGF and 5 ng/ml IL-1β for 6 h and followed by treating cells with IL-1β antibodies and 20 µM cisplatin (CDDP) for 24 h. The apoptotic cells were examined by using Annexin V and PI staining in flow cytometric analysis. The apoptosis ratio was then calculated as shown in (**E**). Values are means ± S.E. of three determinations. n.s.: no significant difference. ***: P<0.005; **: P< 0.01.

To investigate the biological significance of EGF-induced IL-1β expression in tumor cells, we focused on drug resistance in cisplatin-induced apoptosis. As shown in Figure 7A, EGF and IL-1β stimulated cell growth and recovered cisplatin-induced cell death. Based on an examination of dehydrogenase activity in viable cells using CCK-8 assay, the viability of cells also recovered with the addition of EGF and IL-1β in cisplatin-treated cells (Figure 7B). These results suggest that EGF and IL-1β may prevent cell death under a cisplatin-treated condition. The cisplatin-induced activation of caspase 3 was analyzed to further verify that the protection produced by EGF and IL-1β resulted from reducing cisplatin-induced apoptosis. Compared with cisplatin treatment, EGF and IL-1β reduced cisplatin-activated caspase 3 (Figure 7C). These results indicated that EGF and IL-1β protected cells from cisplatin-induced apoptosis. We studied the possibility that EGF-protected cells from cisplatin-induced apoptosis were caused by the induction of IL-1β expression. Using flow cytometry assay, we observed that cisplatin-induced cell apoptosis was reduced considerably in cells treated with EGF and IL-1β(Figure 7D). The depletion of IL-1β with a specific antibody in the cultured media decreased the protection effect of EGF and IL-1β on cisplatin-induced cytotoxicity (Figures 7D and 7E). To further clarify that EGF-induced IL-1β contributed to chemotherapeutic resistance of cancer cells, the expression of IL-1β was knockdown by shIL-1β in oral cancer cells. As shown in Figure S4, EGF protected SCC25 cells from cisplatin-induced apoptosis, however, shIL-1β cells were more sensitive to cisplatin-induced cell death and inhibited EGF protection. These results showed that EGF-induced IL-1β expression protected tumor cells from chemotherapeutic drug-induced cell death.

## Discussion

To our knowledge, this study is the first to show that EGF induces IL-1β expression through the activation of the NF-κB pathway and increased the secretion of IL-1β to protect cancer cells from cisplatin-induced cell cytotoxicity. It is unclear whether IL-1 is overexpressed in tumor cells to mediate the transformation process or generated as a by-product of the transformed cells. However, most studies indicated that autocrine IL-1 production by tumor cells increases invasiveness patterns; anti-tumor effects were rarely observed. For example, IL-1β content correlated with aggressive tumors and modulated the microenvironment to the benefit of tumor growth, invasion, and metastasis by activation of proteolytic enzymes, stroma formation and angiogenesis [32], [33]. These observations are consistent with our results that IL-1β is a protector to regulate EGF-enhanced cell survival when cells experience chemotoxicity. We also found that EGF enhanced the expression of IL-1β and up-regulated the expression of IL-8 and IL-11. Although the mechanisms by which EGF induced the expression of IL-11 to correlate with physiological functions remain to be determined, the EGF-induced secretion of IL-1β may occur through the autocrine circuit to activate the IL-1 receptor, resulting in the secretion of IL-8 or other secondary cytokines that promote tumor cell survival and invasion [34]. Thus, the contribution of growth factor signaling and activated parallel or second waves of cytokine signals to tumorigenesis is a crucial issue.

Two methods are used to control the biosynthesis and secretion of IL-1β, including transcriptional activation of gene expression and activation of the IL-1β-converting enzyme (ICE); therefore the formation and secretion of IL-1β mediates cellular functions, such as inflammation, proliferation and survival. For example, in the regulation of ICE, lipopolysaccharides (LPS) and TNF-α activate ICE to enhance the secretion of IL-1β in monocytic, endothelial, and dendritic cells [35], [36]. In addition, EGF induces the expression and activation of ICE in pancreatic carcinoma cells [37]. Our results also showed that EGF enhanced the expression of IL-1β and the secretion of IL-1β in the cultured media. Thus, EGF may regulate the expression and secretion of IL-1β by activation of transcriptional activity and ICE, respectively. In the control of IL-1β gene expression using several stimulators (PMA, LPS, TNF-α, and TGF-β), enhanced transcription of IL-1β is induced by the binding of multiple transcription factors, such as NF-κB, AP-1, NF-IL6, and CREB, to its promoter [38]. The 3’-UTR region of IL-1β mRNA is also regulated by EGF, resulting in an increase of mRNA stability in human thymic epithelial cells [39]. However, the mechanism involved in the regulation of EGF-induced expression of IL-1β is unclear. To clarify the role of EGF in the regulation of IL-1β expression, we assessed the gene expression and promoter activity of IL-1β in EGF-treated cancer cell lines, such as TU183, CA922, SCC25, SCC4, and A431 cells. Our results showed that EGF-induced expression of IL-1β occurred through the activation of transcriptional activity, but not the stabilization of mRNA. The activation and binding of NF-κB to the IL-1β promoter was essential for EGF-enhanced gene expression. These results show that the EGF-regulated IL-1β occurred through the transcriptional activation in squamous cell carcinomas.

EGF-regulated gene expression is dependent on the activation of downstream effectors of EGFR, such as the PI3/Akt, MEK/ERK, and JAK/STAT signaling pathways. According to our results, Akt/NF-κB was the main pathway for EGF-induced IL-1β gene expression. The JNK and ERK pathways were also activated in cells treated with EGF; however, activation of these pathways was not correlated to the induction of IL-1β expression. These results were consistent with the concept that the activation of the NF-κB pathway is critical for the expression of cytokines (IL-1, IL-2, IL-4, IL-8, and TNF-α) [40]. In addition to the activation of NF-κB being essential for IL-1β gene expression, EGF also regulates IL-6 and IL-8 expression through the activation of STAT-3 and NF-κB, respectively [21], [41]. In addition, it has been reported that IL-6 autocrine activates STAT3 in an EGFR-independent manner and contribute to HNSCC cells proliferation [42], however, we found that IL-6 level was not changed in EGF-treated cells. These results indicated that IL-6 was not involved in EGF-regulated protection of cells from cisplatin-induced cell death. Thus, the activation of EGFR signaling pathways is critical for the expression of specific pro-inflammatory cytokines. These results indicated that the tumorigenecity may be controlled by the regulation of cross-talk between growth and inflammatory signaling pathways.

Although the functional role of IL-1β in the regulation of tumorigenesis, tumor invasiveness, metastasis, and tumor-host interactions has been characterized [12], the main role of EGF-induced IL-1β expression remains unclear. Regarding the correlation between IL-1β and chemotherapy, IL-1β stimulates hematopoiesis to rescue mice after lethal irradiation or chemotherapy [43], [44]. In addition, IL-1β reduces the cisplatin-induced cytotoxicity by stimulating the expression of metallothionein [45]. However, the controversial result shows that the increase of IL-1β enhances cisplatin-induced cell death in brain tumor T98G-resistance cells [46]. These results indicate that the effect of IL-1β on chemotherapy remains unclear, and its role must be further characterized. Future studies will investigate whether the expression of metallothionein is also regulated by EGF-induced IL-1β. In addition, EGF also regulates the chemotherapeutic efficacy of cisplatin. For example, EGF enhances cisplatin-induced ovarian cancer cell apoptosis [47], and the degradation of EGFR is correlated with cisplatin-induced cytotoxicity in head and neck cancers [48]. We also found that EGF protected cells from cisplatin-induced cytotoxicity by inducing IL-1β expression and secretion. It is crucial to determine the manner in which EGF-induced IL-1β regulates cancer cells to escape from cisplatin-induced cytotoxicity. Because both EGF and IL-1β regulate COX-2 expression [20], [49], we propose that IL-1β may cooperate with EGF to induce the expression of COX-2, resulting in enhanced tumorigenesis. Using such a positive feedback circuit, IL-1β may participate in the tumorigenesis by the regulation of PGE_2_ production. The association between COX-2 expression and IL-1β-induced angiogenesis was also studied. In human tumor cell lines grown under normoxic conditions, IL-1β up-regulates the functional HIF-1α protein through a classical inflammatory signaling pathway, involving NF-κB and COX-2, resulting in VEGF secretion by the cancer cells [50]. COX-2-expressing macrophages are essential for IL-1β-induced neovascularization and tumor progression [51]. These results support the proposal that EGF-induced expression of IL-1β and COX-2 may mediate cell inflammation and tumor growth.

In conclusion, this study demonstrated the up-regulation of IL-1β in EGF-treated cancer cells. The expression of IL-1β, at least in part, reduced cisplatin-induced tumor cell death. These results correlated growth factor and inflammatory signaling pathways with drug resistance. In addition to understanding the diverse functions of IL-1β in the interaction between tumor cells and immune response, the production of IL-1β from tumor cells induced by growth factor is a crucial issue for the development of novel therapeutic modalities based on intervention in IL-1β expression. Differential manipulation of IL-1β by EGF in squamous cell carcinomas can offer new methods for targeting IL-1 in cancer therapy.

## Supporting Information

Figure S1 **EGF induces the expression of IL-1β in differential cell lines.** Squamous cell carcinoma TU183, CA922, SCC25, SCC4 cells were treated with 50 ng/ml EGF and 5 ng/ml IL-1β for 6 h before the extraction of RNA. The expression of IL-1β and GAPDH mRNA was analyzed by RT-PCR and examined in 2% agarose gel.
(TIF)

Figure S2 **shRelA and shAkt1 inhibit EGF-induced IL-1β expression in oral cancer cells.** (**A**) and (**B**) The RelA and Akt1 deficient cell lines were selected by infecting oral cancer cells with lentivirus containing an expression vector encoding a short hairpin RNA (shRNA) against RelA (shRelA) and Akt1 (shAkt1). These stable cells with knockdown of RelA and Akt1 were treated with 50 ng/ml EGF for 6 h before the extraction of RNA. The expression of IL-1β, RelA, Akt1 and GAPDH mRNA was analyzed by RT-PCR and examined in 1% agarose gel.
(TIF)

Figure S3 **JNK and MAPK are not involved in EGF-induced IL-1β expression.** (**A**) and (**B**) A431 cells were treated with 20 µM U0126 or 30 µM SP600125 for 1 h, followed by 50 ng/ml EGF treatment for 6 h before the extraction of RNA. The expression of IL-1β and GAPDH mRNA was analyzed by RT-PCR and examined in 2% agarose gel.
(TIF)

Figure S4 **IL-1β knockdown cells are more sensitive to cisplatin treatment.** (**A**) The IL-1β deficient cells were selected by infecting SCC25 cells with lentivirus containing an expression vector encoding a short hairpin RNA (shRNA) against IL-1β (sh IL-1β). The expression of IL-1β and GAPDH mRNA was analyzed by RT-PCR and examined in 2% agarose gel (upper panel). Expression of IL-1β was analyzed by Real-time PCR (lower panel). Values represent means ± S.E. of three independent experiments. (**B**) These stable cells with knockdown of IL-1β were treated with 50 ng/ml EGF for 6 h and followed by treating cells with 40 µM cisplatin (CDDP) for 24 h. The apoptotic cells were examined by using Annexin V and PI staining in flow cytometric analysis. The apoptosis ratio was calculated. *: early apoptosis; #: late apoptosis
(TIF)
