# Supplementary figures and images for: Epidermal Growth Factor Protects Squamous Cell Carcinoma against Cisplatin-Induced Cytotoxicity through Increased Interleukin-1β Expression
Source: PLoS One. 2013 Feb 1;8(2):e55795. doi: 10.1371/journal.pone.0055795 (PMC3562190; doi:10.1371/journal.pone.0055795)

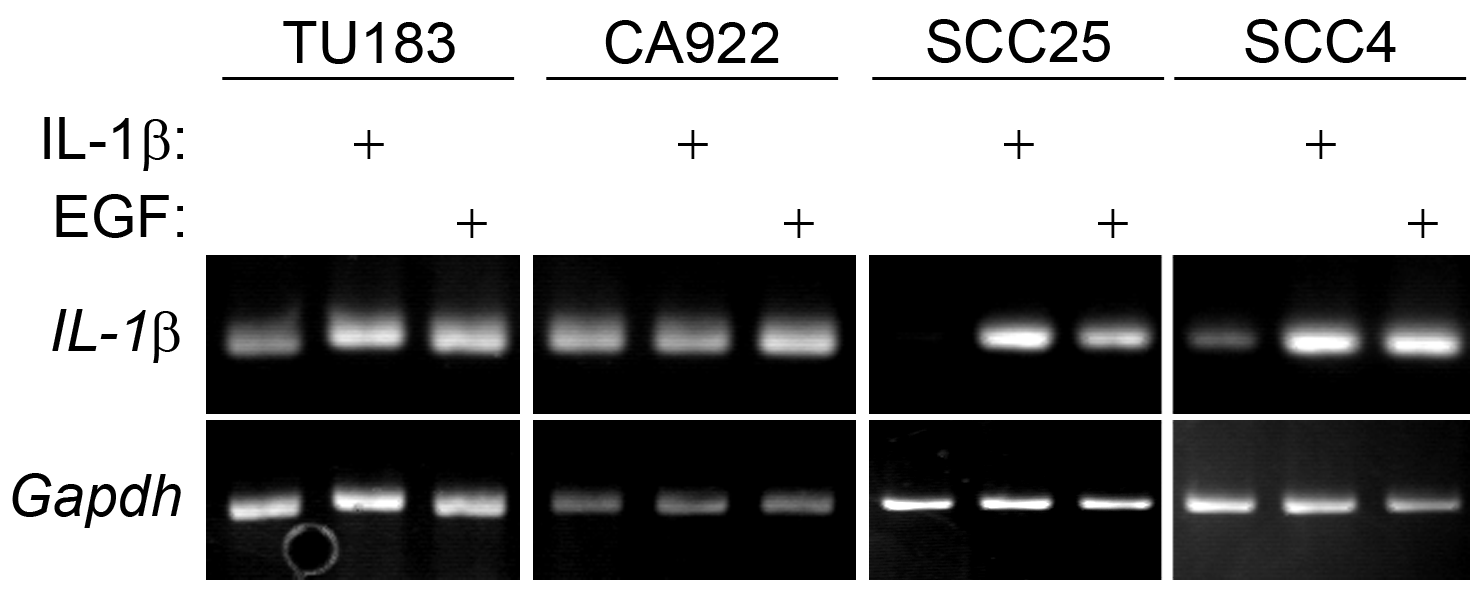

Supplement: Figure S1 — EGF induces the expression of IL-1β in differential cell lines. Squamous cell carcinoma TU183, CA922, SCC25, SCC4 cells were treated with 50 ng/ml EGF and 5 ng/ml IL-1β for 6 h before the extraction of RNA. The expression of IL-1β and GAPDH mRNA was analyzed by RT-PCR and examined in 2% agarose gel. (TIF) [file pone.0055795.s001.tif]

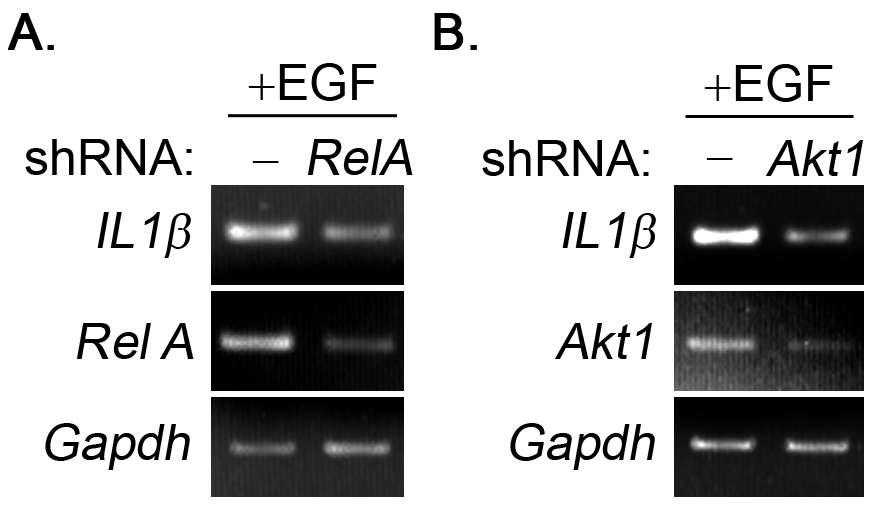

Supplement: Figure S2 — shRelA and shAkt1 inhibit EGF-induced IL-1β expression in oral cancer cells. (A) and (B) The RelA and Akt1 deficient cell lines were selected by infecting oral cancer cells with lentivirus containing an expression vector encoding a short hairpin RNA (shRNA) against RelA (shRelA) and Akt1 (shAkt1). These stable cells with knockdown of RelA and Akt1 were treated with 50 ng/ml EGF for 6 h before the extraction of RNA. The expression of IL-1β, RelA, Akt1 and GAPDH mRNA was analyzed by RT-PCR and examined in 1% agarose gel. (TIF) [file pone.0055795.s002.tif]

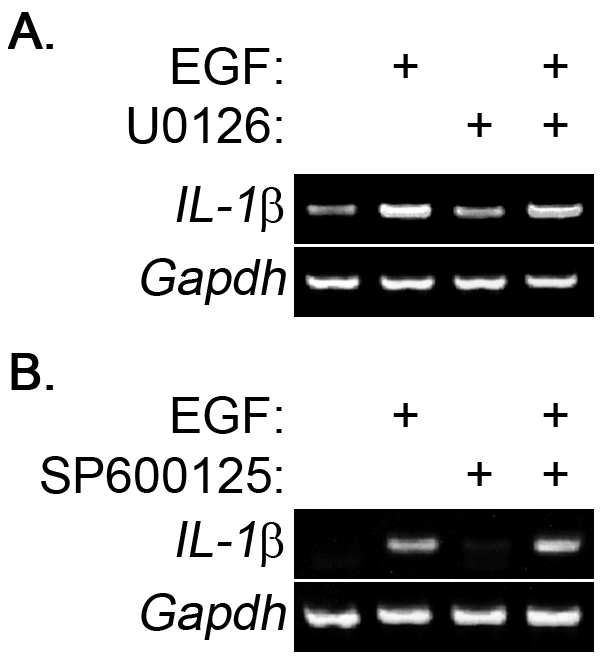

Supplement: Figure S3 — JNK and MAPK are not involved in EGF-induced IL-1β expression. (A) and (B) A431 cells were treated with 20 µM U0126 or 30 µM SP600125 for 1 h, followed by 50 ng/ml EGF treatment for 6 h before the extraction of RNA. The expression of IL-1β and GAPDH mRNA was analyzed by RT-PCR and examined in 2% agarose gel. (TIF) [file pone.0055795.s003.tif]

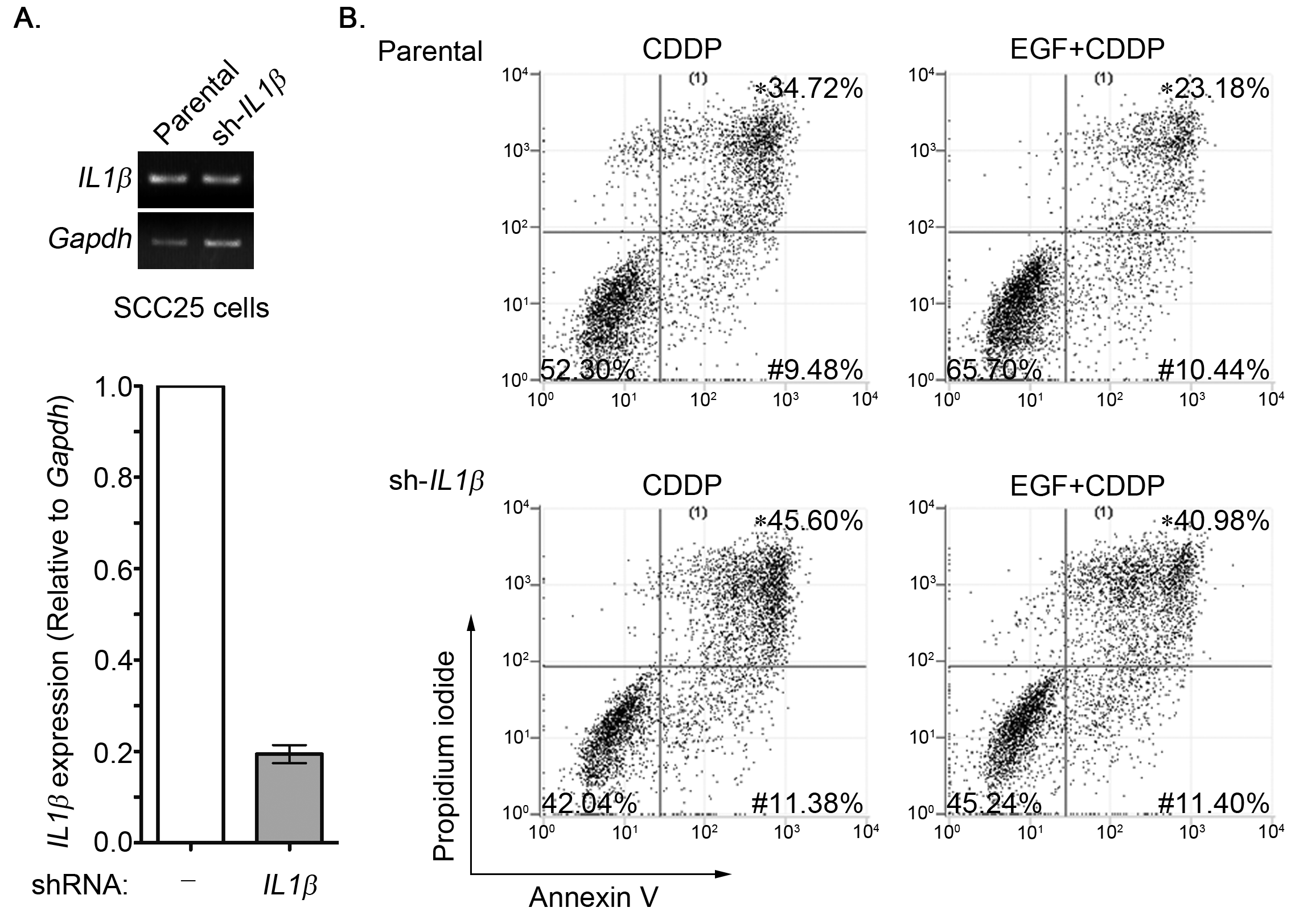

Supplement: Figure S4 — IL-1β knockdown cells are more sensitive to cisplatin treatment. (A) The IL-1β deficient cells were selected by infecting SCC25 cells with lentivirus containing an expression vector encoding a short hairpin RNA (shRNA) against IL-1β (sh IL-1β). The expression of IL-1β and GAPDH mRNA was analyzed by RT-PCR and examined in 2% agarose gel (upper panel). Expression of IL-1β was analyzed by Real-time PCR (lower panel). Values represent means ± S.E. of three independent experiments. (B) These stable cells with knockdown of IL-1β were treated with 50 ng/ml EGF for 6 h and followed by treating cells with 40 µM cisplatin (CDDP) for 24 h. The apoptotic cells were examined by using Annexin V and PI staining in flow cytometric analysis. The apoptosis ratio was calculated. *: early apoptosis; #: late apoptosis (TIF) [file pone.0055795.s004.tif]
